# Assessing ChatGPT-4 as a clinical decision support tool in neuro-oncology radiotherapy: a prospective comparative study

**DOI:** 10.1007/s11060-025-05254-z

**Published:** 2025-10-15

**Authors:** Paolo Tini, Federica Novi, Flavio Donnini, Armando Perrella, Giulio Bagnacci, Maria Antonietta Mazzei, Giuseppe Minniti

**Affiliations:** 1 Unit of Radiation Oncology, Department of Medicine, Surgery and Neurosciences, University of Siena, Siena, Italy; 2 Unit of Diagnostic Imaging, Department of Medicine, Surgery and Neurosciences, University of Siena, Siena, Italy; 3 Radiation Oncology, Policlinico Umberto I, Department of Radiological, Oncological and Pathological Sciences, ″Sapienza″ University of Rome, Rome, Italy; 4 IRCSS Neuromed, Pozzilli, Italy

**Keywords:** ChatGPT-4, Large language model, Neuro-oncology, Radiotherapy, Clinical decision support, Artificial intelligence

## Abstract

**Background and purpose:**

Large language models (LLMs) such as ChatGPT-4 have shown potential for medical decision support, but their reliability in specialized fields remains uncertain. This study aimed to evaluate ChatGPT-4’s performance as a clinical decision support tool in neuro-oncology radiotherapy by comparing its treatment recommendations for patients with central nervous system tumors against a multidisciplinary tumor board’s decisions, an independent specialist’s opinion, and published guidelines.

**Materials and methods:**

We prospectively collected 101 neuro-oncology cases (May 2024–May 2025) presented at a tertiary-care tumor board. Key case details were entered into ChatGPT-4 with a standardized query asking whether to recommend radiotherapy and, if so, the target volumes and dose. The AI’s recommendations were recorded and compared to the tumor board’s consensus, a blinded radiation oncologist’s recommendation, and ESMO guideline indications when applicable. Concordance rates (percentage agreement) and Cohen’s kappa were calculated. Sensitivity and specificity were assessed using the reference decisions as ground truth. McNemar’s test was used to evaluate any bias in discordant recommendations.

**Results:**

ChatGPT-4 matched the tumor board’s radiotherapy recommendations in 76% of cases (κ = 0.61). Agreement with the independent specialist was 79% (κ = 0.58). In 61 low-complexity cases with clear guidelines, ChatGPT-4 concurred with guideline-based indications in 76.7% of cases, missing some recommended treatments (sensitivity 73%, specificity 100%). In intermediate-complexity scenarios, concordance with the tumor board was 70.8%, with most discrepancies due to the AI recommending treatment that experts did not (sensitivity 85.7%, specificity 64.7%). In high-complexity cases, agreement was 90.9% (sensitivity 100%, specificity 83.3%). Overall, ChatGPT-4 showed an *overtreatment bias*, more often recommending radiotherapy when the human experts chose observation (*p* < 0.05 for AI vs. tumor board discordances). Its overall agreement (76%) was lower than that of the human specialist (90%).

**Conclusion:**

ChatGPT-4 can reproduce many expert radiotherapy decisions in neuro-oncology, reflecting substantial absorption of standard clinical practice. However, it cannot substitute for human judgment: the AI omitted some indicated treatments in straightforward cases and suggested unnecessary therapy in some borderline cases, indicating a lack of nuanced clinical reasoning. Careful human oversight is essential if such models are to be used for clinical decision support.

## Introduction

Recent advances in LLMs have raised the prospect of AI-assisted clinical decision support. Models like OpenAI’s ChatGPT have demonstrated impressive breadth of knowledge, even passing standardized exams such as the USMLE medical licensing exam [1] and specialty board questions. Furthermore, studies show that LLMs like ChatGPT can achieve expert-level performance in radiation oncology physics and hold potential for applications in treatment planning [2, 3]. These findings suggest that modern LLMs capture a substantial portion of medical domain knowledge and reasoning. There is growing interest in leveraging such models to aid clinicians – for example, by generating therapeutic recommendations or providing decision support based on clinical guidelines [4, 5]. Radiotherapy planning for neuro-oncology (central nervous system tumors) is a prime example of a high-stakes, knowledge-intensive decision process that might benefit from AI support. Determining whether to recommend radiotherapy after surgery, and if so, defining the treatment volumes and dose, requires integrating evidence-based guidelines with patient-specific factors. While guidelines (e.g. ESMO or NCCN) provide general recommendations, experienced physicians often adjust them based on tumor histology, location, patient performance status, comorbidities, and multidisciplinary consensus [6]. Errors or suboptimal choices in radiotherapy planning can significantly impact patient outcomes and toxicity [7, 8]. An AI tool that reliably reproduces expert recommendations could enhance consistency and provide a “virtual second opinion” in complex or borderline cases.ChatGPT-4, a state-of-the-art general LLM, has already shown the ability to deliver coherent medical advice in general contexts [9]. However, its safety and validity in specialized, complex domains like neuro-oncology remain largely untested. Prior reports note that ChatGPT and similar models may *hallucinate* false information or lack up-to-date clinical knowledge [10]. Moreover, an LLM operates on statistical correlations in text and lacks true understanding of clinical nuance [11]. This raises concern that it might confidently recommend an incorrect or harmful treatment in an ambiguous scenario. In this study, we investigated whether ChatGPT-4 can function as a clinical decision support tool for radiotherapy in neuro-oncology. Specifically, we assessed how well ChatGPT-4’s therapeutic recommendations for brain tumor cases align with those of human experts and guideline indications. Our objectives were to quantify concordance between the AI and established decision sources, evaluate the AI’s accuracy in suggesting appropriate treatment volumes and doses, and identify patterns of discrepancy (such as systematic under- or over-treatment). By analyzing performance across cases of varying complexity, we aim to understand the current limitations of a general-purpose LLM in a highly specialized medical task. We also discuss the implications of our findings for future integration of AI assistants in clinical workflows, particularly in oncology where multidisciplinary collaboration is key.

## Materials and methods

### Study design and cases

We conducted a single-institution prospective observational study comparing ChatGPT-4’s radiotherapy recommendations to expert decisions. The study included 101 consecutive cases of central nervous system tumors discussed at a multidisciplinary neuro-oncology tumor board in a tertiary hospital between May 2024 and May 2025. Each case represented a real patient scenario considered for post-surgery radiotherapy.The distribution of tumor types was as follows (Table 1.: glioblastoma (*n* = 52), meningiomas (*n* = 24), low-grade gliomas (*n* = 15), and other rare primary CNS tumors (e.g., ependymoma, oligodendroglioma, pineal region tumors; *n* = 10). No pediatric cases were included. All cases had sufficient diagnostic and treatment details in the tumor board records; no cases were excluded, in order to capture a full range from straightforward to borderline situations.

**Table 1:** Distribution of tumor types in the study cohort (*n* = 101)

Tumor type	Number of cases (*n*)	Percentage (%)
Glioblastoma (IDH-wild type and mutant)	52	51.5%
Meningioma	24	23.8%
Low-grade gliomas (WHO grade II–III)	15	14.9%
Rare primary CNS tumors*	10	9.9%
**Total**	**101**	**100%**

* Rare tumors included: ependymoma (*n* = 4), oligodendroglioma (*n* = 3), pineal region tumors (*n* = 2), other uncommon histologies (*n* = 1)

### Case complexity classification

**Fig. 1:**
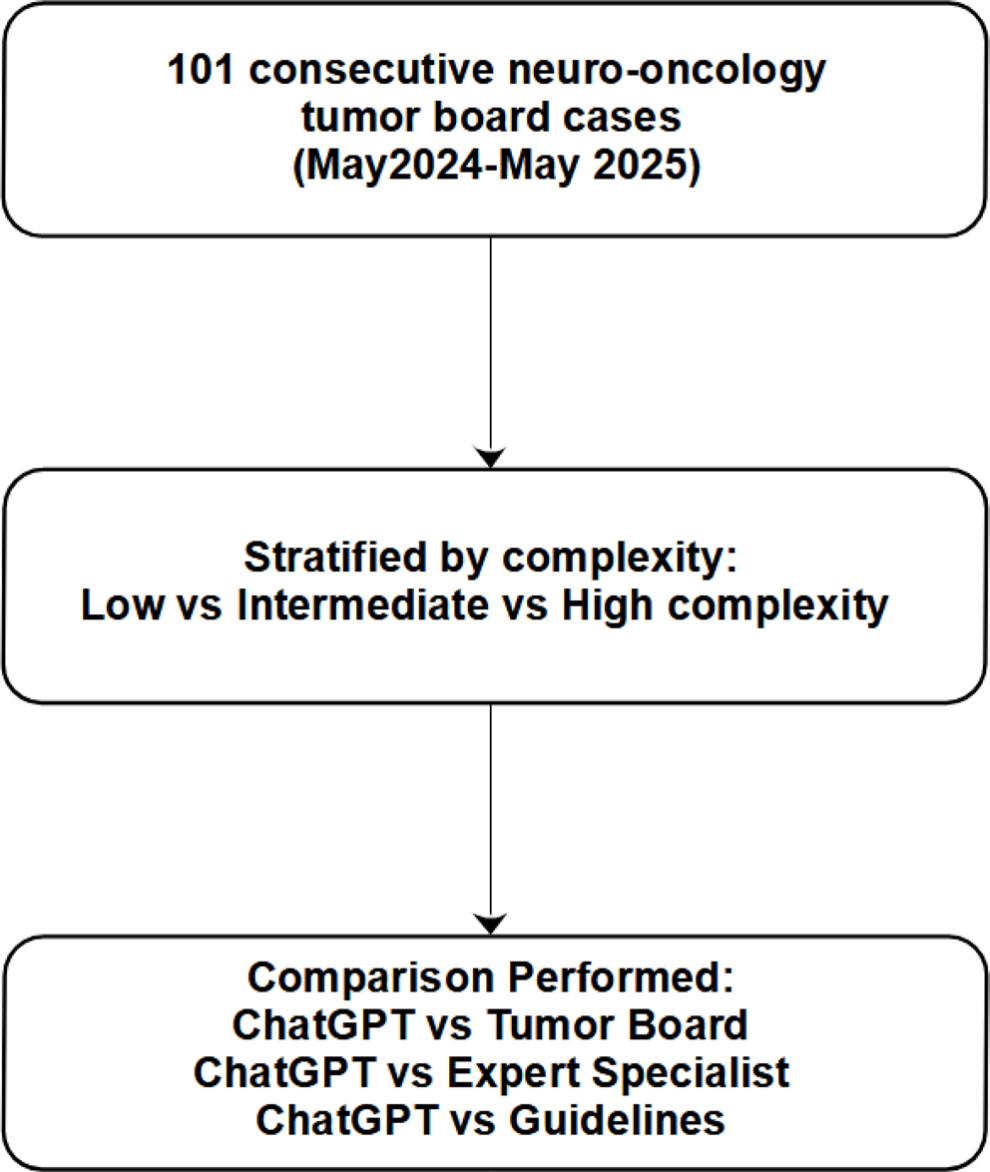
Flow diagram of case selection and analysis. A total of 101 consecutive neuro-oncology tumor board cases (May 2024–May 2025) were assessed, all included and analyzed. Cases were stratified by complexity (low = 61; intermediate = 26; high = 14). Comparisons performed: ChatGPT-4 vs. tumor board (*n* = 101); ChatGPT-4 vs. specialist (*n* = 101); and ChatGPT-4 vs. guidelines (low-complexity only, *n* = 61)

For analysis, each case was classified as *low*, *intermediate*, or *high* complexity based on the availability of clear treatment guidelines (Fig. 1). Low-complexity cases were those with well-established guidelines offering a clear recommendation (e.g. a standard indication for postoperative radiotherapy in a malignant tumor). Intermediate-complexity denoted cases without explicit guidelines but some supporting evidence or partial expert consensus, leading to variability in practice. High-complexity cases had no relevant guidelines and limited or conflicting evidence, requiring highly individualized clinical judgment. This stratification was defined a priori to examine whether ChatGPT-4’s performance varies with case complexity. In addition to complexity-based stratification, we performed a post-hoc exploratory analysis stratified by tumor histology (glioblastoma, meningioma, low-grade gliomas, and other rare primary CNS tumors). The objective was to assess whether ChatGPT-4’s performance varied across different tumor types. For each subgroup, concordance with the tumor board was calculated.

### ChatGPT-4 query procedure

For consistency, all case summaries followed the same structure, including patient age, sex, performance status, histology, prior treatments, and imaging findings. For example, the standardized prompt was: *‘Patient: male, 62 years, ECOG 1, glioblastoma after gross total resection, no prior radiotherapy. MRI shows postoperative cavity without residual disease. Question: would you perform radiotherapy in this case? If yes, specify target volumes and total dose/fractionation.’* (original queries were in Italian, the language of the medical records. We used the GPT-4 model (OpenAI, data cutoff October 2023) via the ChatGPT interface. Each query was started fresh with no chat history carried over between cases. The AI was not given any clarifications or additional information beyond the initial prompt, simulating a single-turn consultation. If the AI’s answer was unclear or noncommittal, no further probing was done. To assess response consistency, we repeated the same query for all cases after a 30-day interval (using the same GPT-4 version) and recorded whether the recommendations changed.

### Reference standard decisions

We collected three comparator decisions for each case to serve as reference points: (1) the multidisciplinary tumor board (MTB) consensus on whether to recommend radiotherapy (and the intended target/dose if yes), as documented during the meeting; (2) an independent, experienced radiation oncologist’s recommendation based on the same case summary provided to ChatGPT (this specialist was not part of the tumor board and was blinded to the board’s and AI’s decisions); and (3) the recommendation according to published clinical guidelines (specifically, the European Society for Medical Oncology – ESMO – guidelines) for that scenario, if applicable [12–15]. We selected ESMO guidelines as the primary standard for low-complexity cases. Concordance was cross-checked qualitatively with ESTRO and EANO guidelines, which showed > 95% overlap with ESMO for the included scenarios. This choice ensured consistency and alignment with European practice. In low-complexity cases, guidelines typically provide a clear “yes or no” on radiotherapy, which we used as the reference. In intermediate or high-complexity cases where no specific guideline exists, a guideline reference was considered not applicable. (We chose ESMO guidelines as a consistent standard-of-care reference; other guideline sets like NCCN were noted qualitatively but not formally applied.)

### Outcome measures

The primary outcome was agreement between ChatGPT-4 and each reference decision on whether radiotherapy was recommended. For each case we determined whether ChatGPT-4’s binary recommendation (yes or no for radiotherapy) matched the decision of the reference source. We calculated the raw concordance rate (percentage of cases with agreement) and Cohen’s κ statistic to account for chance agreement. This analysis was done for three comparisons: ChatGPT vs. the tumor board (all 101 cases), ChatGPT vs. the independent specialist (101 cases), and ChatGPT vs. guidelines (for the 61 low-complexity cases where guidelines provided a recommendation). For context, we also computed the concordance between the independent specialist and the tumor board. Additionally, within each complexity subgroup, we evaluated ChatGPT’s performance relative to the designated reference standard for that subgroup. Treating the human or guideline decision as the “ground truth” for needing radiotherapy, we calculated ChatGPT’s sensitivity (the fraction of cases that *required* radiotherapy in which the AI also recommended treatment) and specificity (the fraction of cases that did *not* require radiotherapy where the AI correctly recommended omission of treatment). These metrics characterize the AI’s tendency toward false negatives (missing a needed treatment) or false positives (recommending an unnecessary treatment) in each scenario. Beyond the binary decision, we assessed the quality of ChatGPT’s treatment plan details. In cases where both ChatGPT and the human expert(s) recommended radiotherapy, we compared the technical specifics. We noted whether ChatGPT’s suggested radiation target volumes matched the physician’s intended treatment field (e.g. the same tumor bed or regions at risk) and whether the proposed dose/fractionation was within a reasonable range of the expert’s prescription. We tallied the percentage of cases with a matching target and a matching dose/fractionation between the AI and the human plan.

### Statistical analysis

Agreement statistics (concordance rates and κ) were interpreted using standard benchmarks (κ values of 0.41–0.60 indicating moderate agreement, 0.61–0.80 substantial, > 0.80 near-perfect). We used McNemar’s chi-square test for paired binary outcomes to examine whether discordant decisions were biased in a particular direction (i.e., whether ChatGPT was more likely to say “yes” when the reference said “no” or vice versa). This test was applied to each comparison (overall and within subgroups). We also directly compared ChatGPT’s agreement with the tumor board to the specialist’s agreement with the tumor board using a paired analysis across the 101 cases. Sensitivity (ability to recommend indicated radiotherapy) and specificity (ability to correctly withhold radiotherapy when not indicated) were calculated for each subgroup, treating the reference standard decision as ground truth. For these proportions, 95% confidence intervals were estimated using the binomial exact (Clopper–Pearson) method to quantify statistical uncertainty. A two-sided p-value < 0.05 was considered statistically significant for all analyses. Data were analyzed using SPSS (v28).

## Results

### Overall concordance

All patients underwent the treatment as decided by the multidisciplinary tumor board. Therefore, the tumor board decision represented both the clinical reference standard and the treatment actually delivered in all cases. ChatGPT-4’s recommendation on radiotherapy matched the multidisciplinary tumor board’s decision in 76% of cases (κ = 0.606, indicating substantial agreement). It agreed with the independent specialist in 79% of cases (79/101, κ = 0.583). In the 61 low-complexity cases with clear guideline indications, ChatGPT aligned with ESMO guideline recommendations in 76.7% of cases (κ = 0.420, indicating moderate agreement). By comparison, the independent specialist agreed with the tumor board in 89.9% of cases. The gap between ChatGPT’s and the specialist’s concordance with the board was statistically significant (76% vs 89.9%, p = 0.004), The concordance between the independent specialist and the tumor board was 90% (κ = 0.72). This indicates that intra-human agreement remains superior to AI–human agreement.” (Table 2).

**Table 2:** Overall agreement between ChatGPT-4 and reference decision sources on radiotherapy recommendation (*n* = 101 cases). *Guideline comparison is evaluated only for low-complexity cases where an established guideline recommendation exists

Comparison	Concordance (%)	Cohen’s κ
ChatGPT-4 vs. Tumor Board (*n* = 101)	76.0%	0.606
ChatGPT-4 vs. Specialist (*n* = 101)	79.0%	0.583
ChatGPT-4 vs. Guidelines (*n* = 61)*	76.7%	0.420
Specialist vs. Tumor Board (*n* = 101)	90.0%	0.720

### Performance by case complexity

**Fig. 2:**
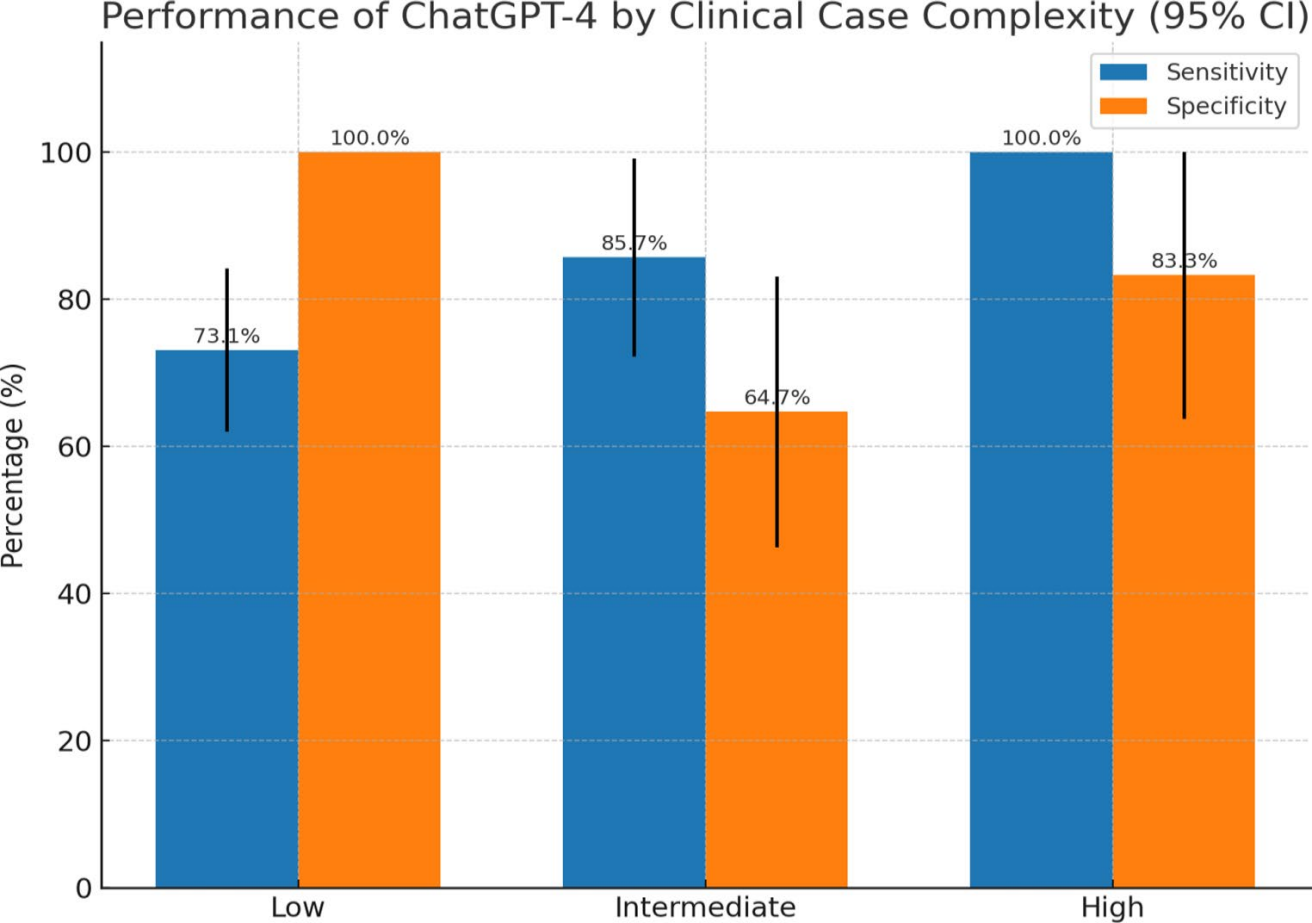
Performance of ChatGPT-4 by clinical case complexity. Bars show sensitivity (ability to recommend indicated radiotherapy) and specificity (ability to correctly withhold radiotherapy when not indicated) for low-, intermediate-, and high-complexity cases. In low-complexity cases, the model never suggested unwarranted therapy (specificity 100%) but missed some indicated treatments (sensitivity 73.1%; 95%-CI: 62.0–84.2%). In intermediate cases, sensitivity remained high (85.7%, 95%-CI: 72.2–99.2%) while specificity declined (64.7%, 95%-CI: 46.3–83.1%), reflecting a propensity to overtreat. In high-complexity cases, both sensitivity (100%) and specificity (83.3%, 95% - CI: 63.8–100%) were high. Error bars represent 95% confidence intervals

As shown in Table 3; Fig. 2, in low-complexity scenarios ChatGPT concurred with guideline-based treatment indications in 76.7% of cases (κ = 0.420, moderate agreement). All 14 discordant low-complexity cases were instances where the AI failed to recommend radiotherapy despite a guideline indication – McNemar’s test confirmed a significant asymmetry (*p* = 0.0005), with ChatGPT systematically missing guideline-indicated treatments (undertreatment bias). In intermediate-complexity cases, concordance with the tumor board was 70.8% (κ = 0.417, moderate agreement). Most disagreements in this group were due to ChatGPT recommending treatment that the experts did not, suggesting an *overtreatment* tendency (sensitivity 85.7%, specificity 64.7%, *p* = 0.13 for bias toward recommending therapy). In high-complexity cases, ChatGPT achieved 90.9% agreement with the tumor board (κ = 0.820, indicating near-perfect agreement), with only 1 discordant case out of 11 analyzed. In that single high-complexity discordance, the AI recommended treatment where the board did not (sensitivity 100%, specificity 83.3%). Thus, the AI’s highest performance was observed in the most complex scenarios. When stratified by histology (Table 4), ChatGPT-4 achieved the highest concordance in glioblastoma and other high-grade gliomas (82%). In contrast, concordance was lower in meningiomas and low-grade gliomas (70 and 71%), without significant differences between these groups. These findings suggest that the model reproduces guideline-based decisions more reliably in tumors with standardized radiotherapy indications, such as high-grade gliomas.

**Table 3:** ChatGPT-4 performance by clinical case complexity

Complexity (*n* cases)	Reference standard for “truth”	Concordance (%)	Cohen’s κ
Low (61 cases)	Guidelines (ESMO)	76.7%	0.420
Intermediate (26 cases)	Tumor Board	70.8%	0.417
High (14 cases)	Tumor Board	90.9%	0.820

**Table 4:** Concordance between ChatGPT-4 and the tumor board stratified by histology

Tumor type	Number of cases (*n*)	Concordance with tumor board (%)	Cohen’s κ
Glioblastoma & other HGG	52	82.0%	0.67
Low-grade gliomas	15	71.0%	0.52
Meningiomas	24	70.0%	0.50
Rare primary CNS tumors*	10	72.0%	0.51
**Total**	**101**	**76.0%**	**0.61**

* Rare tumors included ependymoma, oligodendroglioma, pineal region tumors and others histologies

### Treatment plan details

We next examined the concordance in technical planning specifics. In 45 cases where both ChatGPT-4 and a human expert recommended radiotherapy, the AI’s suggested target volumes matched the physician’s actual treatment field in 66.7% of instances. Similarly, ChatGPT’s proposed total dose and fractionation scheme was within an acceptable range of the expert’s prescription in 81.8% of 44 evaluable cases (one case lacked a clearly defined dose in the AI response). In the remaining cases, the AI either omitted a target that the physician included or suggested a dose outside the typical range used by the expert. In summary, when ChatGPT did recommend radiotherapy correctly, its technical plan fully aligned with the human plan roughly two-thirds of the time, and the dose/fractionation was appropriate about 82% of the time.

### Discordance bias

Overall, when ChatGPT disagreed with the tumor board’s decision (in 24 of 101 cases), it was usually by *recommending* treatment that the board did not. Specifically, 75% of AI vs. board discordances were of this type (18/24 cases; McNemar *p* = 0.014), indicating a significant overtreatment bias overall. In practical terms, this bias reflects ChatGPT’s tendency to recommend radiotherapy in situations where experts opted for observation, such as small asymptomatic meningiomas in elderly patients. A similar pattern was observed in comparison to the specialist’s decisions (McNemar *p* = 0.029 for ChatGPT vs. specialist, again reflecting the AI’s tendency to recommend more therapy). By contrast, the tumor board versus guideline comparison revealed that the human experts often chose to omit radiotherapy in situations where guidelines would have recommended it (McNemar *p* < 0.0001), reflecting the experts’ tendency to individualize treatment beyond one-size-fits-all guidelines.

### Reproducibility

ChatGPT-4’s recommendations were highly reproducible over time. When all case queries were repeated after 30 days, the model gave the same yes/no recommendation in 99% of cases (100 out of 101), corresponding to a test–retest κ = 0.96. This indicates excellent internal consistency of the AI outputs, i.e. the model’s advice did not drift over the short term.

## Discussion

This prospective study demonstrates that ChatGPT-4 can reproduce a substantial portion of expert reasoning in neuro-oncology radiotherapy decisions, yet it also highlights significant limitations. Concordance rates of approximately 76–79% against physicians and guidelines indicate that the model has indeed absorbed much of standard oncology practice, consistent with prior reports of LLMs performing well on medical tasks [16–18]. Importantly, the concordance between the independent specialist and the tumor board was 90% (κ = 0.72), clearly higher than ChatGPT’s concordance with the tumor board. This highlights that intra-human agreement remains superior to AI–human agreement, underscoring the current gap between large language models and experienced clinicians. Compared to established clinical decision-support systems (CDSS) such as IBM Watson for Oncology or OncoKB, ChatGPT-4 differs fundamentally in design and operation. Traditional CDSS tools are guideline-driven and provide transparent, rule-based recommendations but have limited adaptability beyond predefined clinical pathways. In contrast, ChatGPT is a general-purpose large language model that generates fluent recommendations by leveraging statistical associations in text. In our study, this resulted in high reproducibility (99%), but at the expense of explainability and a tendency toward systematic biases (overtreatment bias in gray-zone scenarios). These differences highlight both the promise and the limitations of LLMs compared with existing decision-support platforms, and underscore the need for human oversight.

The notably high agreement in very complex cases suggests that an AI might serve as a knowledgeable assistant in decision-making for scenarios where clinicians rely on general principles and available evidence. However, the AI’s significantly lower agreement compared to a human specialist (76% vs. 90%) confirms it cannot match an experienced clinician. In roughly 1 out of 4 cases overall, ChatGPT’s recommendation differed from the expert consensus, which is consistent with previous papers showing current LLMs falling short of expert-level decision agreement [19]. The exploratory histology-specific analysis confirmed that ChatGPT-4 aligned best with expert decisions in glioblastoma and other high-grade gliomas, where radiotherapy indications are well established. Conversely, in meningiomas and low-grade gliomas, concordance was lower and relatively similar across histologies, reflecting the greater variability of clinical decision-making in these settings (e.g., observation vs. postoperative RT in WHO grade I–II tumors). This suggests that the model is more reliable when applied to tumor types with clear guideline-based standards of care, while its performance decreases in entities characterized by individualized or heterogeneous practice patterns.

A key insight is that ChatGPT’s performance varied with case context. In straightforward, guideline-defined scenarios, the AI was relatively conservative – it *missed* some treatments that guidelines would have recommended (i.e. undertreatment). This may reflect that ChatGPT is not simply regurgitating guidelines but has “learned” that clinicians sometimes deviate (for instance, sparing certain patients from therapy despite a formal indication). Conversely, in intermediate “gray zone” scenarios lacking clear rules, ChatGPT tended to over-intervene, essentially adopting a “when in doubt, treat” approach.The interpretation of κ values further contextualizes these findings. While κ around 0.42 (moderate agreement) in guideline-defined cases signals non-negligible discordance, κ > 0.80 (near-perfect) in high-complexity scenarios may reflect both a small sample effect and shared default strategies between experts and AI. Importantly, McNemar’s test highlighted that these discordances were systematically biased rather than random. Concrete examples from our dataset illustrate the clinical implications of these systematic biases. Undertreatment errors, although less frequent than overtreatment, may carry a disproportionately greater clinical risk. For instance, ChatGPT did not recommend adjuvant radiotherapy in a newly diagnosed glioblastoma patient after gross total resection, despite a clear guideline indication—an omission that could compromise survival. By contrast, an overtreatment example occurred in an 80-year-old frail patient with an incidental meningioma, where ChatGPT recommended radiotherapy while the tumor board opted for observation to avoid unnecessary toxicity. While undertreatment may be more detrimental clinically, the overall pattern of discordance shows that ChatGPT exhibited a global tendency to recommend radiotherapy more often than human experts. This overtreatment tendency could stem from its training on medical literature that often emphasizes active treatment. In the most complex cases, both human experts and the AI defaulted to aggressive management; here the model’s behavior happened to align with expert instinct (treat when options are limited), resulting in its highest concordance. These findings must be interpreted cautiously because the high-complexity subgroup was small (*n* = 14). This limited sample size inflates κ values and sensitivity/specificity estimates, increasing the risk of type II error and limiting the precision of the reported performance.

Notably, agreement with experts does not guarantee optimal care. Our comparison of tumor board decisions to guidelines showed that even human experts deviate from guidelines to individualize care [20]. ChatGPT’s biases seem to echo these human deviations, but without real clinical understanding [11]. Essentially, the AI mirrors patterns in its training data: its undertreatment of some low-complexity cases likely corresponds to scenarios in which prudent physicians chose observation over therapy (e.g. frail patients or minimal residual disease), while its overtreatment bias in other cases reflects an inclination toward intervention drawn from the literature [21]. Unlike a human oncologist, however, ChatGPT cannot account for critical patient-specific nuances – such as age, comorbidities, or personal values – nor can it seek clarification on ambiguous details. This limits its ability to make truly context-aware decisions.

Our findings underscore the need for human oversight when using such AI tools. The model’s errors illustrate potential hazards if its suggestions were followed uncritically. For example, ChatGPT failed to recommend re-irradiation in a case of aggressive tumor recurrence (which could have led to undertreatment), and it suggested aggressive therapy for an elderly patient that experts managed with observation (which could have caused unnecessary harm). These examples show how the AI, lacking holistic clinical context, can misjudge situations that physicians navigate by weighing risks, benefits, and patient preferences.

Looking ahead, ChatGPT-4 should be viewed as a support tool rather than a decision-maker [22]. In its current form, it may be useful for providing a quick second opinion or summarizing guideline-based options – for instance, helping a trainee double-check standard recommendations – but any AI suggestion must be validated by clinicians. Specialized medical LLMs fine-tuned on oncology-specific data might achieve better accuracy in the future, but they will require extensive prospective validation [23]. Ultimately, the appropriate role of AI is to *augment* human decision-making, not replace it. Our results support a model of human–AI collaboration: the AI can rapidly provide evidence-based insights and consistency checks, while human experts apply the nuanced judgment and ethical considerations that remain uniquely their domain. Embracing such collaboration cautiously could enhance decision-making in neuro-oncology, provided we remain aware of the AI’s limitations and maintain the primacy of human clinical judgment.

## Conclusion

ChatGPT-4 was able to mirror standard radiotherapy decision-making in a majority of neuro-oncology cases, demonstrating that LLMs can encapsulate many clinical guidelines and practices. However, it also exhibited notable biases, tending to undertreat in some guideline-defined cases and overtreat in ambiguous ones. These limitations mean that while ChatGPT-4 (and similar AI) may serve as useful decision support tools, they cannot replace expert clinical judgment. Careful integration – with clinicians double-checking AI recommendations – will be essential if such models are to be used safely in practice. Further refinement of medical LLMs and thorough validation studies are needed before routine clinical deployment.

## Data Availability

No datasets were generated or analysed during the current study.

## References

[1] Kung TH, Cheatham M, Medenilla A, Sillos C, De Leon L, Elepaño C, Madriaga M, Aggabao R, Diaz-Candido G, Maningo J, Tseng V (2023) Performance of ChatGPT on USMLE: potential for AI-assisted medical education using large Language models. PLOS Digit Health 2(2):e0000198. 10.1371/journal.pdig.0000198PMID: 36812645; PMCID: PMC9931230 36812645 10.1371/journal.pdig.0000198 PMC9931230

[2] Wang P, Holmes J, Liu Z, Chen D, Liu T, Shen J, Liu W. A recent evaluation on the performance of LLMs on radiation oncology physics using questions of randomly shuffled options. Front Oncol; 15:1557064 10.3389/fonc.2025.1557064 PMC12141255 40485729

[3] Schramm F, Haggenmüller S, Kather JN, Hetz MJ, Wies C, Michel MS, Wessels F, Brinker TJ (2025) Large language model use in clinical oncology. NPJ Precis Oncol. 2024;8(1):240. doi: 10.1038/s41698-024-00733-4. PMID: 39443582; PMCID: PMC11499929 10.1038/s41698-024-00733-4 PMC11499929 39443582

[4] Hao Y, Qiu Z, Holmes J et al (2025) Large Language model integrations in cancer decision-making: a systematic review and meta-analysis. Npj Digit Med 8:450. 10.1038/s41746-025-01824-7 40676129 10.1038/s41746-025-01824-7 PMC12271406

[5] Ramadan S, Mutsaers A, Chen PC, Bauman G, Velker V, Ahmad B, Arifin AJ, Nguyen TK, Palma D, Goodman CD (2025) Evaluating chatgpt’s competency in radiation oncology: A comprehensive assessment across clinical scenarios. Radiother Oncol 202:110645 Epub 2024 Nov 19. PMID: 39571686 39571686 10.1016/j.radonc.2024.110645

[6] Vinod SK, Merie R, Harden S (2025) Quality of decision making in radiation oncology. Clin oncol (R coll Radiol). 38:103523. 10.1016/j.clon.2024.02.001. Epub 2024 Feb 6. PMID: 38342658 10.1016/j.clon.2024.02.001 38342658

[7] Moore KL, Schmidt R, Moiseenko V, Olsen LA, Tan J, Xiao Y, Galvin J, Pugh S, Seider MJ, Dicker AP, Bosch W, Michalski J, Mutic S (2015) Quantifying unnecessary normal tissue complication risks due to suboptimal planning: A secondary study of RTOG 0126. Int J Radiat Oncol Biol Phys 92(2):228–235 Epub 2015 Apr 3. PMID: 25847605; PMCID: PMC4431941 25847605 10.1016/j.ijrobp.2015.01.046 PMC4431941

[8] Ohri N, Shen X, Dicker AP, Doyle LA, Harrison AS, Showalter TN (2013) Radiotherapy protocol deviations and clinical outcomes: a meta-analysis of cooperative group clinical trials. J Natl Cancer Inst 105(6):387–393. 10.1093/jnci/djt001Epub 2013 Mar 6. PMID: 23468460; PMCID: PMC3601950 23468460 10.1093/jnci/djt001 PMC3601950

[9] Chuang WK, Kao YS, Liu YT, Lee CY Assessing ChatGPT for Clinical Decision-Making in Radiation Oncology, With Open-Ended Questions and Images. Pract Radiat Oncol. 2025 Apr 29:S1879-8500(25)00115-8. 10.1016/j.prro.2025.04.009. Epub ahead of print. PMID: 40311921 10.1016/j.prro.2025.04.009 40311921

[10] Deng J, Zubair A, Park YJ, Affan E, Zuo QK (2024) The use of large language models in medicine: proceeding with caution. Curr Med Res Opin. ;40(2):151–153. doi: 10.1080/03007995.2023.2295411. Epub 2024 Jan 24. PMID: 38093584 10.1080/03007995.2023.2295411 38093584

[11] Griot M, Hemptinne C, Vanderdonckt J, Yuksel D (2025) Large Language models lack essential metacognition for reliable medical reasoning. Nat Commun 16(1):642. 10.1038/s41467-024-55628-6PMID: 39809759; PMCID: PMC11733150 39809759 10.1038/s41467-024-55628-6 PMC11733150

[12] Le Rhun E, Guckenberger M, Smits M, Dummer R, Bachelot T, Sahm F, Galldiks N, de Azambuja E, Berghoff AS, Metellus P, Peters S, Hong YK, Winkler F, Schadendorf D, van den Bent M, Seoane J, Stahel R, Minniti G, Wesseling P, Weller M, Preusser M, EANO Executive Board and ESMO Guidelines Committee (2021) Electronic address: clinicalguidelines@esmo.org. EANO-ESMO clinical practice guidelines for diagnosis, treatment and follow-up of patients with brain metastasis from solid tumours. Ann Oncol 32(11):1332–1347. 10.1016/j.annonc.2021.07.016Epub 2021 Aug 6. PMID: 34364998 34364998 10.1016/j.annonc.2021.07.016

[13] Stupp R, Brada M, van den Bent MJ, Tonn JC, Pentheroudakis G, ESMO Guidelines Working Group (2014) High-grade glioma: ESMO clinical practice guidelines for diagnosis, treatment and follow-up. Ann Oncol 25(Suppl 3):iii93–101. 10.1093/annonc/mdu050Epub 2014 Apr 29. PMID: 24782454 24782454 10.1093/annonc/mdu050

[14] Le Rhun E, Weller M, van den Bent M, Brandsma D, Furtner J, Rudà R, Schadendorf D, Seoane J, Tonn JC, Wesseling P, Wick W, Minniti G, Peters S, Curigliano G, Preusser M, EANO Guidelines Committee and ESMO Guidelines Committee (2023) Electronic address: clinicalguidelines@esmo.org. Leptomeningeal metastasis from solid tumours: EANO-ESMO clinical practice guideline for diagnosis, treatment and follow-up. ESMO Open 8(5):101624. 10.1016/j.esmoop.2023.101624Epub 2023 Sep 19. PMID: 37863528; PMCID: PMC10619142 37863528 10.1016/j.esmoop.2023.101624 PMC10619142

[15] Niyazi M, Andratschke N, Bendszus M, Chalmers AJ, Erridge SC, Galldiks N, Lagerwaard FJ, Navarria P, Munck Af Rosenschöld P, Ricardi U, van den Bent MJ, Weller M, Belka C, Minniti G (2023) ESTRO-EANO guideline on target delineation and radiotherapy details for glioblastoma. Radiother Oncol 184:109663 Epub 2023 Apr 13. PMID: 37059335 37059335 10.1016/j.radonc.2023.109663

[16] Shool S, Adimi S, Saboori Amleshi R, Bitaraf E, Golpira R, Tara M (2025) A systematic review of large Language model (LLM) evaluations in clinical medicine. BMC Med Inf Decis Mak 25(1):117. 10.1186/s12911-025-02954-4PMID: 40055694; PMCID: PMC11889796 10.1186/s12911-025-02954-4 PMC11889796 40055694

[17] Liu C, Liu Z, Holmes J, Zhang L, Zhang L, Ding Y, Shu P, Wu Z, Dai H, Li Y, Shen D, Liu N, Li Q, Li X, Zhu D, Liu T, Liu W (2023) Artificial general intelligence for radiation oncology. Meta Radiol 1(3):100045 Epub 2023 Nov 24. PMID: 38344271; PMCID: PMC10857824 38344271 10.1016/j.metrad.2023.100045 PMC10857824

[18] Li J, Dada A, Puladi B, Kleesiek J, Egger J (2024) ChatGPT in healthcare: A taxonomy and systematic review. Comput Methods Programs Biomed 245:108013. 10.1016/j.cmpb.2024.108013Epub 2024 Jan 15. PMID: 38262126 38262126 10.1016/j.cmpb.2024.108013

[19] Hager P, Jungmann F, Holland R, Bhagat K, Hubrecht I, Knauer M, Vielhauer J, Makowski M, Braren R, Kaissis G, Rueckert D (2024) Evaluation and mitigation of the limitations of large Language models in clinical decision-making. Nat Med 30(9):2613–2622. 10.1038/s41591-024-03097-1Epub 2024 Jul 4. PMID: 38965432; PMCID: PMC11405275 38965432 10.1038/s41591-024-03097-1 PMC11405275

[20] Salloch S, Otte I, Reinacher-Schick A, Vollmann J (2018) What does physicians’ clinical expertise contribute to oncologic decision-making? A qualitative interview study. J Eval Clin Pract 24(1):180–186. 10.1111/jep.12840Epub 2017 Oct 27. PMID: 29076629 29076629 10.1111/jep.12840

[21] Omar M, Soffer S, Agbareia R, Bragazzi NL, Apakama DU, Horowitz CR, Charney AW, Freeman R, Kummer B, Glicksberg BS, Nadkarni GN, Klang E (2025) Sociodemographic biases in medical decision making by large Language models. Nat Med 31(6):1873–1881. 10.1038/s41591-025-03626-6Epub 2025 Apr 7. PMID: 40195448 40195448 10.1038/s41591-025-03626-6

[22] Li J, Zhou Z, Lyu H, Wang Z (2025) Large Language models-powered clinical decision support: enhancing or replacing human expertise? Intell Med 5(1):1–4. 10.1016/j.imed.2025.01.001

[23] Holmes J, Liu Z, Zhang L, Ding Y, Sio TT, McGee LA, Ashman JB, Li X, Liu T, Shen J, Liu W (2023) Evaluating large Language models on a highly-specialized topic, radiation oncology physics. Front Oncol 13:1219326 PMID: 37529688; PMCID: PMC10388568 37529688 10.3389/fonc.2023.1219326 PMC10388568

